# Characterization of *Plasmodium falciparum* Pantothenate Kinase and Identification of Its Inhibitors From Natural Products

**DOI:** 10.3389/fcimb.2021.639065

**Published:** 2021-03-09

**Authors:** Arif Nurkanto, Ghulam Jeelani, Herbert J. Santos, Yulia Rahmawati, Mihoko Mori, Yumi Nakamura, Kana Goto, Yoko Saikawa, Takeshi Annoura, Yuzuru Tozawa, Takaya Sakura, Daniel Ken Inaoka, Kazuro Shiomi, Tomoyoshi Nozaki

**Affiliations:** ^1^ Research Center for Biology, Indonesian Institute of Sciences (LIPI), Cibinong, Indonesia; ^2^ Department of Biomedical Chemistry, Graduate School of Medicine, The University of Tokyo, Tokyo, Japan; ^3^ Kitasato Institute for Life Sciences, Kitasato University, Tokyo, Japan; ^4^ Biological Resource Center, National Institute of Technology and Evaluation (NITE), Chiba, Japan; ^5^ Department of Applied Chemistry, Faculty of Science and Technology, Keio University, Yokohama, Japan; ^6^ Department of Parasitology, National Institute of Infectious Diseases (NIID), Tokyo, Japan; ^7^ Graduate School of Science and Engineering, Saitama University, Saitama, Japan; ^8^ Department of Molecular Infection Dynamics, School of Tropical Medicine and Global Health, Institute of Tropical Medicine (NEKKEN), Nagasaki University, Nagasaki, Japan

**Keywords:** coenzyme A, *Pf*PanK1, *Pf*PanK2, *Plasmodium falciparum*, inhibitors

## Abstract

Coenzyme A (CoA) is a well-known cofactor that plays an essential role in many metabolic reactions in all organisms. In *Plasmodium falciparum*, the most deadly among *Plasmodium* species that cause malaria, CoA and its biosynthetic pathway have been proven to be indispensable. The first and rate-limiting reaction in the CoA biosynthetic pathway is catalyzed by two putative pantothenate kinases (*Pf*PanK1 and 2) in this parasite. Here we produced, purified, and biochemically characterized recombinant *Pf*PanK1 for the first time. *Pf*PanK1 showed activity using pantetheine besides pantothenate, as the primary substrate, indicating that CoA biosynthesis in the blood stage of *P. falciparum* can bypass pantothenate. We further developed a robust and reliable screening system to identify inhibitors using recombinant *Pf*PanK1 and identified four *Pf*PanK inhibitors from natural compounds.

## Introduction

Malaria is an infectious disease in humans caused by *Plasmodium* species and transmitted through the bites of female *Anopheles* mosquitoes. Among five *Plasmodium* species, *P. falciparum*, causes the highest mortality worldwide, and is responsible for 99.7% cases in Africa, 50% in South-Eastern Asia, 71% in the Eastern Mediterranean, and 65% in the Western Pacific. It is estimated that this parasite infects more than 200 million people and is responsible for 445,000 deaths in 2017 (WHO Report, 2017), with the number being slightly decreased to 405,000 in 2018 (WHO, 2019). Although many preventive and therapeutic efforts combating malaria helped in reducing its mortality in the last decade, the emergence and spread of antimalarial resistance does not seem to cease. Currently, artemisinin combination therapies (ACTs) are recommended as the standard first-line treatment for uncomplicated falciparum malaria (Visser et al., 2014) to repress malarial drug resistance. However, ACTs resistance were also reported in both Asian (Dondorp et al., 2009) and African countries (Lu et al., 2017).

A number of antimalarial drug candidates recently entered the preclinical and clinical trial stages. Some of them have been proven to have novel targets, while the precise mechanism of action of other candidates remains unknown (Wells et al., 2015; Tse et al., 2019). Current anti-malarial chemotherapy is based on standard therapies in which an artemisinin derivative and its partner drug with a preferably synergistic mechanism are used in combination. However, new antimalarials that are safe, effective with a single oral administration, have a novel mechanism of action with no cross-resistance to existing antimalarials, and active against multiple life stages including both asexual erythrocytic and liver stages in humans and sexual stages in the mosquito, with anti-relapse (*P. vivax*) and transmission-blocking activities (Fidock, 2010; Grimberg and Mehlotra, 2011; Burrows et al., 2017; Tse et al., 2019; Favuzza et al., 2020) are still needed.

Coenzyme A (CoA) is an essential cofactor in many metabolic processes, involving more than 9% of approximately 3,500 cellular activities (www.brenda-enzymes.info/). CoA biosynthesis has been attracting attention as a promising drug target (Fletcher et al., 2016). In *P. falciparum*, CoA is generated by four enzymatic reactions initiated by the conversion of pantothenate (Vitamin B5) to 4-phosphopantothenate, catalyzed by pantothenate kinase (*Pf*PanK) (Saliba and Kirk, 2001). Other steps are mediated by phosphopantothenoylcysteine synthetase (*Pf*PPCS)-phosphopantothenoylcysteine decarboxylase (*Pf*PPCDC) bifunctional enzyme, phosphopantetheine adenylyltransferase (*Pf*PPAT), and dephospho-CoA kinase (*Pf*DPCK), with the last enzyme catalyzing the rate limiting step (Spry et al., 2008). Most prokaryotes and some eukaryotes including mammals and plants are reported to produce pantothenate *de novo* (Begley et al., 2001; Chakauya et al., 2008; Spry et al., 2008). In *Plasmodium*, it has been shown that pantothenate cannot be synthesized *de novo*, thus it is only obtained from the host (Spry et al., 2008; Spry and Saliba, 2009; Spry et al., 2010). The blood stage parasites of *P. falciparum* are absolutely dependent on pantothenate from human plasma or the culture medium (Divo et al., 1985; Mamoun et al., 2010; Augagneur et al., 2013; Bobenchik et al., 2013). In the whole CoA biosynthetic pathway, *Pf*PanK has been best studied in *P. falciparum* (Saliba et al., 1998; Spry et al., 2008). All inhibitors against *Pf*PanK that were so far identified are structurally related to pantothenate, including pantothenamide metabolites (Schalkwijk et al., 2019), pantothenol (PanOH), and CJ-15,801 (Saliba et al., 2005; Spry et al., 2005). Although these potential pantothenate analogs were presumed to target the CoA pathway, they apparently showed no direct inhibition against *Pf*PanK and likely inhibit other targets such as *Pf*PPCS and CoA-utilizing enzymes (Tjhin et al., 2018). Thus, no *Pf*PanK specific inhibitors have been discovered until now.

Although PanK activity was previously measured in lysates from the *P. falciparum* blood stage parasites (Saliba et al., 2005; Spry et al., 2005; Spry et al., 2018; Tjhin et al., 2018), no previous report has described successful expression of bacterial recombinant *Pf*PanK (Hart et al., 2016). In this study, we have developed the overexpression and purification of active *Pf*PanK using an *Escherichia coli* expression system, and biochemically characterized *Pf*PanK. We have also developed a robust enzymatic screening system using recombinant *Pf*PanK and screened the natural compounds from microbes and plants against *Pf*PanK to successfully identify inhibitors with structures unrelated to pantothenate.

## Materials and Methods

### Organism, Chemicals, and Supplies

*Escherichia coli* BL21Star™ (DE3) strain was purchased from Invitrogen (Carlsbad, CA, USA). All chemicals of analytical grade were purchased from Wako (Tokyo, Japan) or Sigma-Aldrich (Tokyo, Japan) unless otherwise stated. Magnesium-free ATP was purchased from DiscoverX (Fremont, CA, USA). Ni^2+^-NTA agarose was purchased from Novagen (Darmstadt, Germany). Structurally elucidated natural products with known efficacy. Gnetin A, gnetin C, and gnemonoside D were gift from Dr. Azuma Watanabe, AM, Fujisawa, Japan. Non-binding, 96-well microplates were purchased from Greiner Bio-One (Frickenhausen, Germany). *Plasmodium falciparum* sensitive strain 3D7 used in asexual blood stage phenotypic assay. AlbuMAX II was purchased from Gibco (Life Technologies, Carlsbad, CA, USA), hypoxanthine (Sigma), sodium l-lactate and nitro-tetrazolium blue chloride (NBT) (Fujifilm, Wako), APAD (Oriental Yeast, Japan).

### Phylogenetic Analyses of *P. falciparum* Pantothenate Kinase

PanK protein sequences (38 orthologues) from representative bacterial and eukaryotic taxa were retrieved from the non-redundant protein sequences (nr) database of the National Center for Biotechnology Information (NCBI, http://www.ncbi.nlm.nih.gov/). As a query, we used the *Pf*PanK1 (PF3D7_1420600/XP_001348373) and only the sequences with an E-value lower than 1 x 10^-10^ were selected. Sequences were aligned using the Muscle program (Edgar, 2004) in SeaView package version 4.6.1 (Gouy et al., 2010). To select hit amino acid sequences for the model, the data matrices for phylogeny were subjected to the IQTREE program (Nguyen et al., 2015). The maximum likelihood (ML) analysis implemented in the RAxML program version 7.2.6 (Stamatakis, 2006) was used to infer ML tree. Bootstrap values higher than 50 (in percentages) are indicated on the corresponding internal branches of the ML tree constructed using FigTree program Version 1.4.2 (http://tree.bio.ed.ac.uk/software/figtree/).

### Expression and Purification of Recombinant *Pf*PanK

The genes encoding two putative PanKs, *Pf*PanK1 and *Pf*PanK2, were synthesized commercially after codon codon optimization for expression in *E. coli* or wheat-germ cell-free system, respectively (Eurofins Genomics, Tokyo, Japan). The *E. coli* codon-optimized *Pf*PanK1 gene (NCBI accession number: MW331581) was inserted into the plasmid pCold™1 histidine-tag vector (Takara, Tokyo, Japan) to produce pCold™1-*Pf*PanK1. The plasmid was introduced to BL21Star™ (DE3) chemically competent cells (ThermoFisher Scientific, Waltham, MA, USA) and the transformed *E. coli* strains were cultured at 37°C in Luria Bertani medium (Invitrogen) in the presence of 100 μg/ml ampicillin (Nacalai Tesque). The overnight culture was used to inoculate 1 L of freshly prepared medium. The culture was further incubated with 100 μg/ml ampicillin at 37°C with shaking at 180 rpm. Isopropyl β-D-thio galactopyranoside (IPTG) was added to the culture at the final concentration of 0.5 mM when A_600_ reached 0.8. Cultivation was continued for another 24 h at 15°C. *E. coli* cells were then harvested by centrifugation at 5,000 x g for 20 min at 4°C. The cell pellet collected was re-suspended in 40 mL of lysis buffer (50 mM Tris HCl, pH 8.0, 300 mM NaCl, and 10 mM imidazole) containing 0.1% Triton X-100 (v/v), 0.7 M trehalose, 100 μg/ml lysozyme, and 1 mM phenylmethylsulfonyl fluoride (PMSF), and incubated at room temperature for 30 min. The cell suspension was then passed through a French press (Ohtake, Tokyo) with high pressure at 800 kg/cm^2^ and centrifuged at 25,000 x g for 30 min at 4°C. The supernatant obtained was mixed with 2 ml of 50% Ni^2+^-NTA His-bind slurry (Qiagen, Germany) then incubated at 4°C with mild shaking for 1 h. The resin with bound recombinant enzyme was washed three times with 50 mM Tris-HCl, pH 8.0, 300 mM NaCl, containing 20 mM imidazole and 0.1% (v/v) Triton X-100. The bound enzyme was eluted with buffer containing stepwise gradient concentrations of imidazole (20–300 mM). The purity of the recombinant protein was confirmed with 12% SDS-PAGE analysis, followed by Coomassie Brilliant Blue (CBB) staining. A fraction containing the pure enzyme was dialyzed against a 300-fold volume buffer containing 50 mM Tris-HCl pH 8.0, 150 mM NaCl, containing 10% glycerol (v/v) supplemented with Complete Mini protease inhibitor cocktail (Roche, Mannheim, Germany) at 4°C for 18 h to remove imidazole. The enzyme was stored at -80°C with 20% glycerol in small aliquots until use. The wheat codon optimized *Pf*PanK2 synthetic gene was cloned into pYT08 vector as previously described (Nozawa et al., 2007) to produce pY08-*Pf*PanK2. Protein was expressed using the TnT^®^ SP6 High-Yield Wheat Germ Protein Expression System (Promega) according to the manufacturer’s instructions. Since no tag was added to *Pf*PanK2 in pYT08-*Pf*PanK2, PanK activity for *Pf*PanK2 was measured in the protein mixture of the wheat germ expression system.

### PanK Enzymatic Assay

PanK activities of *Pf*PanK1 and *Pf*PanK2 were estimated by measuring ADP production with a coupling assay using the ADP Hunter™ Plus Assay kit (DiscoverX, US) according to the manufacturer’s instructions. The assay mixture contained 15 mM HEPES, 20 mM NaCl, 10 mM MgCl_2_, 1 mM EGTA, 0.02% Tween 20, 0.1 mg/ml β-globulin, 2 mM pantothenante, 0.1 mM ATP, and 2.5 µg/ml of *Pf*PanK recombinant enzyme in a 25 µl reaction mixture. Fluorescence intensities were measured to estimate the formation of resorufin at 37°C by excitation at 540 nm and emission at 590 nm. Kinetic data were estimated by curve fitting with the Michaelis–Menten equation using GraphPad Prism (GraphPad Software Inc., San Diego, USA). This experiment was performed in triplicate and kinetic values are presented as the means ± S.E. for three independent assays.

### Screening of Natural Compounds for *Pf*PanK Inhibitors

We screened 247 compounds from the Kitasato Natural Products Library (Mori et al., 2015; Nurkanto et al., 2018) against *Pf*PanK recombinant enzyme. Compounds were dissolved in 50% DMSO at a final concentration of 1 mg/ml. Enzymatic reactions were carried out on a black 96-well microtiter plate with a 20 µl reaction mixture composed of 19 μL enzyme mix (50 μM pantothenate, 60 μM ATP, 50 ng of *Pf*PanK recombinant enzyme in kinase buffer, described previously, and 1 μl of the individual compounds (final concentration 50 µg/ml) at 37°C for 2 h. ADP production was measured using the ADP Hunter™ Plus kinase assay kit, as described above. The inhibition constant was measured in triplicate. Compounds that showed > 50% inhibition at the primary screening were re-tested to confirm that they did not inhibit the enzyme in the coupled assay (pyruvate kinase, pyruvate oxidase, and peroxidase). Compounds that did not inhibit enzymes in the coupled assay were further subjected to the determination of the concentration showing 50% inhibition of PanK1 activity (IC_50_).

### Determination of *P. falciparum* IC50 From Natural Products

Selected hit compounds against *Pf*PanK1 were also tested against *P. falciparum* cells. Before using, parasite culture was synchronized with 5% (w/v) d-sorbitol, as previously described (Lambros and Vanderberg, 1979). Ring stage 0.3% parasitemia (25 µl/well) were placed in a 384-well plate. Serial dilutions (50, 25, 12.5, 6.3, 3.1, 1.6, 0.8, and 0.4 μM) of each compound were used for calculating IC_50_. As the negative growth control, 50 µM of Mefloquine and 20 µM of Atovaquone were used. After 72 h of incubation, parasite growth was determined by diaphorase-coupled lactate dehydrogenase (LDH) assay, as previously described (Hartuti et al., 2018). Each well’s absorbance was measured at 650 nm using SpectraMax Paradigm^®^Multi-Mode microplate reader (Molecular Devices, San Jose, CA, USA). The IC_50_ values were analyzed and calculated with GraphPad PRISM 8.0 (San Diego, California USA).

## Results

### Gene Survey and Identification of *Pf*PanK

Two genes that potentially encode PanK were identified in the genome database of *P. falciparum* 3D7 strain (http://PlasmoDB.org) [PF3D7_1420600 (*Pf*PanK1) and PF3D7_1437400 (*Pf*PanK2)]. The two genes contain open reading frames of 1,560 and 2,301 bp in length, which are presumed to encode 519- and 766-amino acid long proteins with the calculated molecular mass of 59.9 and 91.1 kDa, respectively. These two proteins show only 21% mutual identity. While *Pf*PanK1 harbors all the signatures of eukaryotic PanKs for which enzymatic activity has been demonstrated, *Pf*PanK2 lacks some critical amino acid residues implicated for PanK activity. *Pf*PanK1 only shares 25%–28% positional amino acid identity to human PanK1-4 with the highest similarity to PanK3 (28% identity). *Pf*PanK1 appears to be highly conserved among the Apicomplexa (**Figure S1**), whereas *Pf*PanK2 may represent a divergent member as *Pf*PanK2 is well separated from other PanKs by phylogenetic analysis and shares only a few motifs including DXXVXDXYGX and GLXXXXXASXFG (X is any amino acid) with PanKs from human and plants (Hart et al., 2016). These data, together with the lack of enzymatic activity (see below), indicate that *Pf*PanK2 may be involved in a reaction other than that catalyzed by authentic PanK.

### Expression and Purification of Recombinant *Pf*PanK

*Pf*PanK1 was expressed using *E. coli* expression system with a standard protocol (Fig S2A), but mostly in an insoluble form. After optimization of expression vectors, extraction buffers, detergents, and stabilizing additives, recombinant *Pf*PanK1 became partially soluble with a supplementation of 0.7 M trehalose in the extraction buffer (Fig S2B). The homogeneity of purified recombinant *Pf*PanK1 of an estimated size of 62.5 kDa (59.9 kDa plus a 2.6 kDa histidine tag at the amino terminus) was confirmed and its purity was estimated to be >95%, as evaluated with SDS-PAGE gel followed by Coomassie Brilliant Blue staining (**Figure S3A**, **Table S1**) and immunoblot analysis using anti-histidine tag antibody (**Figure S3B**). The specific activity of *Pf*PanK1 was estimated to be 9.6 µmole/min/mg (**Table S1**) when assayed under the standard conditions. *Pf*PanK1 was catalytically active in a broad pH range with maximum activity obtained at pH 8.4 and 37°C (**Figure S4A**). On the other hand, production of recombinant *Pf*PanK2 using *E. coli* expression system was unsuccessful. Instead, *Pf*PanK2 was successfully expressed using wheat germ expression system (**Figure S5A**). Since *Pf*PanK2 was not fused with the histidine tag, we attempted to measure activity in the crude lysate but no activity was detected (**Figure S5B**). Although there is a possibility that the produced *Pf*PanK2 was improperly folded or truncated, which likely resulted in loss or reduction of activity, our data are consistent with the premise that *Pf*PanK1, but not *Pf*PanK2, is a functional enzyme, as previously suggested (Tjhin et al., 2018). Therefore, our downstream research was conducted only with *Pf*PanK1.

### Phosphoryl Donor Specificity and Metal Ion Requirement of *Pf*PanK1 Activity

*Pf*PanK1 can catalyze phosphorylation of both pantothenate and pantetheine using ATP as a phosphoryl donor. The kinetic parameters such as *K*_m_, *V*_max_, and *k*_cat_ values for *Pf*PanK1 using pantothenate, pantetheine, and ATP as substrates were determined (**Tables 1** and **2**). *Pf*PanK1 exhibited hyperbolic saturation kinetics when assayed over the substrate range of 4-128 μM for pantothenate with a saturated concentration (120 µM) of ATP (**Figure S6A**) and with 1-200 μM ATP and the saturated concentration (100 µM) of pantothenate (**Figure S6B**). Similar profiles were also obtained when pantetheine and ATP were used (**Figures S6C, D**, respectively). The apparent *K*m values for pantothenate and ATP were 44.5 ± 5.5 and 59.2 ± 15.9 μM, respectively (**Table 1**). Similarly, the *K*m values for pantetheine and ATP were 45.7 ± 6.9 and 43.4 ± 3.3 μM, respectively.

**Table 1:** Kinetic parameters of *P. falciparum* pantothenate kinase 1 with pantothenante, pantetheine, and ATP.

Substrate	*K*_m_ (µM)	*V*_max_ (µmole/min/mg)	*k*_cat_ (min^-1^)	*k*_cat_/*K*_m_ (min^-1^µM^-1^)
Pantothenate	44.6 ± 5.6	14.2 ± 0.71	854 ± 77	19.1 ± 1.0
ATP	59.3 ± 7.9	15.9 ± 2.5	966 ± 103	16.1 ± 1.0
Pantetheine	45.7 ± 6.9	19.6 ± 1.2	1171 ± 25	25.6 ± 0.7
ATP	43.4 ± 3.4	18.3 ± 3.0	1233 ± 54	26.6 ± 6.3

Mean ± SEM are shown.

**Table 2:** Phosphoryl donor specificity of *P. falciparum* pantothenate kinase 1.

NTP^*^	Relative activity (%)	
	Pantothenate	Pantetheine
ATP	100	100
GTP	107.6 ± 8.1	106.5 ± 4.4
UTP	64.1 ± 1.0	23.8 ± 2.3
CTP	28.1 ± 2.8	11.3 ± 0.6
TTP	13.1 ± 1.0	0.0 ± 0.0
dATP	9.0 ± 2.2	8.5 ± 2.7
None	0.0 ± 0.0	0.0 ± 0.0

Assays were performed as described in Materials and methods, in the presence 15 mM HEPES, pH 8.4, 20 mM NaCl, 10 mM MgCl_2_, 1 mM EGTA, 0.02% Tween-20, 0.1 mg/ml β−globulins and 0.2 mM pantothenate. Reactions were conducted at 37°C.

^*^The final concentration used was 100 µM.

The activity is shown in percentage (%) relative to that toward ATP.

Mean ± SEM are shown.

*Pf*PanK1 utilizes various nucleoside triphosphates such as ATP, CTP, GTP, UTP, TTP, and dATP as a phosphate donor (**Table 2**). *Pf*PanK1 showed a slightly higher activity with GTP compared to ATP. *Pf*PanK1 showed an absolute requirement for a free divalent metal cofactor, with Mg^2+^ as the preferred cation for reactions using either pantothenate or pantetheine (**Table 3**). Ferrous cation supported a comparative activity with Mg^2+^ for phosphorylation of pantothenate, but approximately 60% of activity for phosphorylation of pantetheine. Other cations showed lower activity (**Table 3**). No significant difference was observed in the preference on nucleoside phosphates and metals between the reactions where pantothenate or pantetheine was used as a substrate.

**Table 3:** Effect of metal ions on the activity of *P. falciparum* pantothenate kinase 1.

Metal ions*	Relative activity (%)	
	Pantothenate	Pantetheine
MgCl_2_	100	100
FeCl_2_	104.7 ± 14.9	58.3 ± 3.3
NiCl_2_	49.0 ± 5.5	41.4 ± 3.4
MnCl_2_	41.7 ± 8.4	55.3 ± 0.1
NaCl	41.4 ± 5.3	33.1 ± 6.2
CaCl_2_	39.9 ± 4.7	30.5 ± 1.6
LiCl_2_	37.9 ± 9.6	32.6 ± 4.1
ZnCl_2_	35.6 ± 5.6	29.0 ± 0.6
KCl	29.7 ± 2.3	33.6 ± 3.4
CoCl_2_	25.8 ± 1.9	22.3 ± 1.0
CuCl_2_	16.4 ± 1.5	8.5 ± 1.3
none	0.0 ± 0.0	0.0 ± 0.0

Assays were performed as described in Materials and methods, in the presence of 15 mM HEPES, pH 8.4, 20 mM NaCl, 1 mM EGTA, 0.02% Tween-20, 0.1 mg/ml β−globulins, 100 mM ATP, and 0.2 mM pantothenate at 37°C.

^*^The cation final concentration used was 5 mM.

The activity is shown in percentage (%) relative to that toward MgCl_2_.

Mean ± SEM are shown.

### Regulation of *Pf*PanK1 by Coenzyme A, Acetyl CoA, and Panthenol

**Figure 1:**
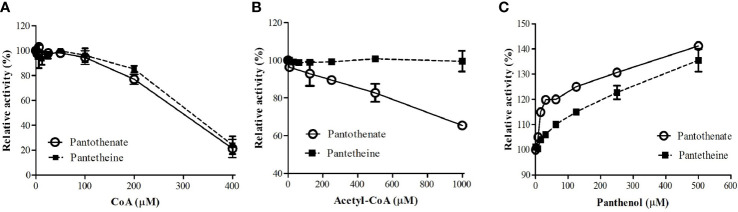
Effect of CoA **(A)**, acetyl CoA **(B)**, and panthenol **(C)** on *P. falciparum* pantothenate kinase activities. Relative activities of *Pf*PanK1 in the presence of various concentrations of inhibitors to those without inhibitors are shown. The assay was performed in 15 mM HEPES, pH 8.4, 10 mM MgCl_2_, 20 mM NaCl, 1 mM EGTA, 0.02% Tween-20, 0.1 mg/ml γ−globulins, 0.2 mM pantothenate, and 100 μM ATP with various concentrations of CoA, acetyl CoA, or panthenol at 37°C. The assay was carried out three times independently, and the results are shown as means ± SEM of triplicates.

It was reported that PanK from other organisms were subjected to regulation by allosteric inhibition with CoA, acetyl CoA, and malonyl CoA (Vallari et al., 1987; Calder et al., 1999; Takagi et al., 2010; Nurkanto et al., 2018). We examined if CoA and acetyl CoA inhibit *Pf*PanK1 activity. *Pf*PanK1 was inhibited by CoA in the presence of pantothenate or pantetheine as a substrate (**Figure 1A**). Acetyl CoA also inhibited *Pf*PanK1 only when pantothenate was used as a substrate; however, relatively higher concentrations were needed (IC_50_ > 1 mM). However, acetyl CoA did not affect *Pf*PanK1 activity when pantetheine was used (**Figure 1B**). In contrast, panthenol, a pantothenate analog, did not inhibit *Pf*PanK1 activity regardless of the substrates, instead high concentrations of panthenol (e.g., 0.5 mM) slightly (up to 40%) increased enzyme activity (**Figure 1C**).

### Identification of *Pf*PanK1 Inhibitors From Natural Product Compounds

**Figure 2:**
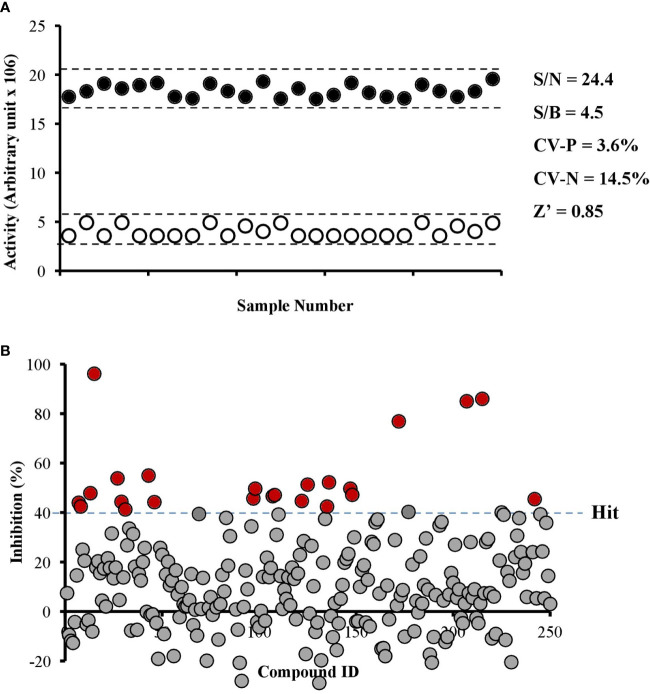
Robustness of *Pf*PanK1 enzymatic assay used in the study **(A)** and an overview of *Pf*PanK1 inhibition by 247 natural compounds **(B)**. **(A)** Determination of Z-factor (Z’), signal-to-noise (S/N), signal-to-background ratio (S/B) and coefficient of variation in positive and negative (CV-P and CV-N, respectively) values of *Pf*PanK1 assay on the 96-well plate format. Solid black and open white circles represent positive and negative (without *Pf*PanK1) reaction, respectively enzyme. **(B)** Scatter plot of the percentage inhibition values of all compounds against *Pf*PanK1 in the primary screening assay. Red solid circles indicate compounds showing >40% inhibition in both the primary and reconfirmation assays.

**Figure 3:**
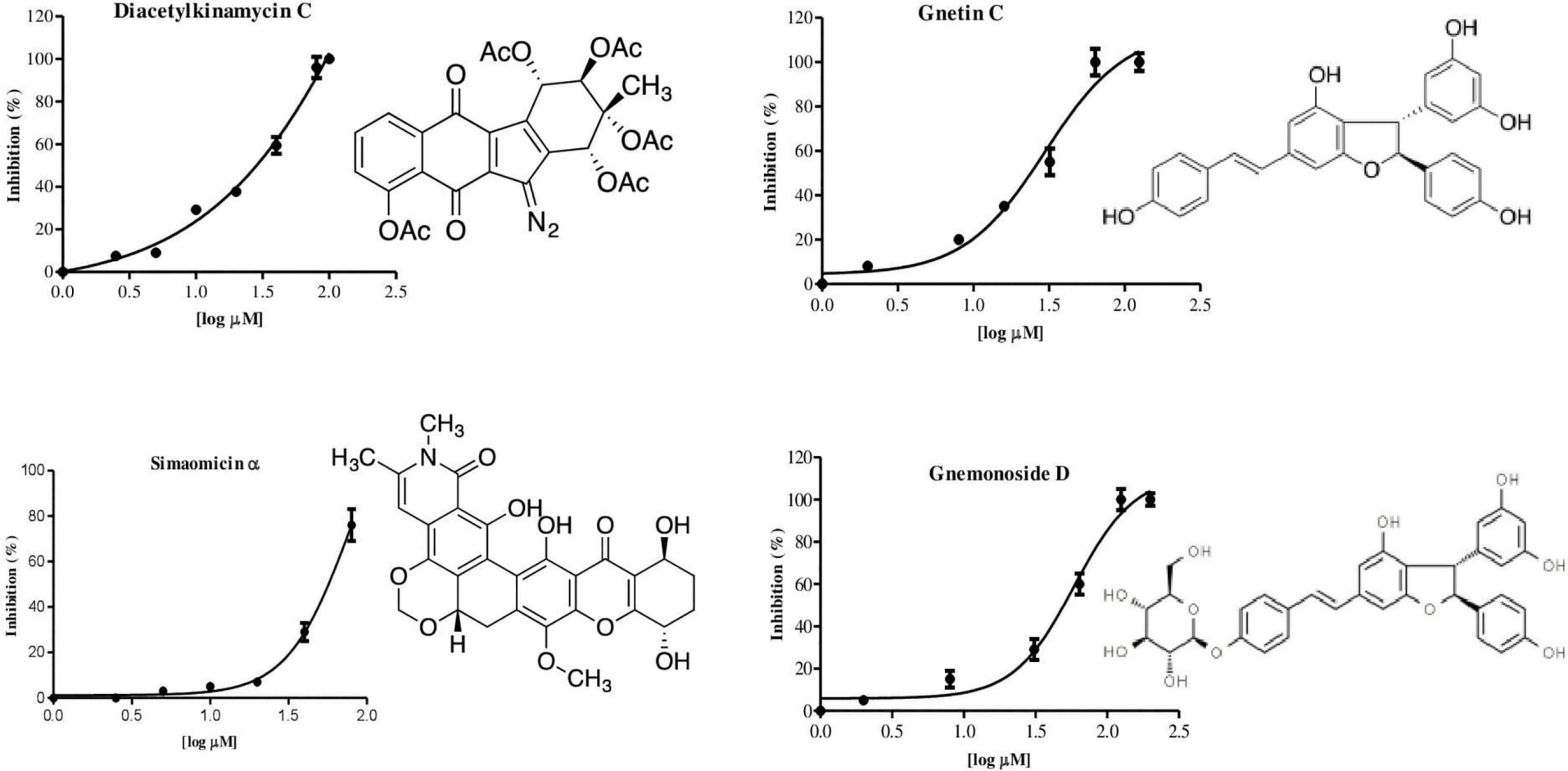
Chemical structure and dose-dependent inhibition for IC_50_ determination of four identified *Pf*PanK1 inhibitors.

We developed an enzyme-based assay using recombinant *Pf*PanK1, to identify its inhibitors by screening chemical and extract libraries. Our assay was proven to be highly sensitive and reproducible with Z’-factor (Zhang et al., 1999) being 0.85 and the signal-to-background ratio of 4.5 (**Figure 2A**). We screened 247 structurally elucidated natural products of Kitasato Natural Compound Library and plant origin. Twenty-five compounds showed > 40% inhibition against *Pf*PanK1 at 50 µM final concentration (**Figure 2B**, **Table S2**). After eliminating the compounds that inhibited the coupling enzyme and those that failed to show inhibition in the reconfirmation assay, four compounds showed dose-dependent inhibition against *Pf*PanK1 (**Figure 3**). These four *Pf*PanK1 inhibitors, gnetin C, diacetylkinamycin C, gnemonoside D, and simaomicin α showed IC_50_ value of 20.3 ± 2.2, 36.2 ± 4.7, 57.5 ± 3.6, and 57.6 ± 4.8 µM, respectively (**Figure 3**, **Table 4**).

**Table 4:** Inhibitory activity (IC_50_ values) of four identified *Pf*PanK1 inhibitors against *Pf*PanK, *P. falciparum* erythrocytic culture, and human cell lines.

Compound	IC_50_ or EC_50_ (µM^$^)		
	*Pf*PanK1	*P. falciparum* erythrocytic stages	Human cell lines
Diacetylkinamycin C	36.2 ± 4.7	2.2 and 1.1^*^	NR
Simaomicin α	57.6 ± 4.8	0.083 and 0.018 nM^**^	0.3–19 nM^#^
Gnetin C	20.3 ± 2.2	12.8 ± 1.9	85–87^‡^
Gnemonoside D	57.5 ± 3.6	1.9 ± 0.6	NR

^$^unless otherwise stated.

^*^The IC_50_ values against P. falciparum K1 and FCR3 strain, respectively (Shimizu el al., 2019, unpublished).

^**^The IC_50_ values against P. falciparum K1 and FCR3 strain (Ui et al., 2007).

^#^The EC_50_ values against various human cell lines (Koizumi et al., 2009).

^‡^The EC_50_ values against human embryonic kidney (HEK-293T) and prostate (RWPE-1) cell lines (Narayanan et al., 2015).

NR, not reported.

Mean ± SEM are shown.

### Growth Inhibition of *Pf*PanK1 Inhibitors Against the *P. falciparum* Erythrocytic Stage Parasites

Two *Pf*PanK1 inhibitors were further tested for their inhibitory activity to the *P. falciparum* erythrocytic stage parasites. Gnetin C and Gnemonoside D showed the IC_50_ values of 12.8 ± 1.9 and 1.9 ± 0.6 µM, respectively. It was previously shown that diacetylkinamycin C showed the IC_50_ values of 1.1–2.2 µM against *P. falciparum* drug-resistant K1 and drug-sensitive FCR3 strain (Shimizu et al., 2019; unpublished). Simaomicin α, been reported has very potent anti-plasmodial activity with the IC_50_ value was 0.083 and 0.018 nM against K1 and FCR3 strains (Ui et al., 2007). Therefore, four compounds that inhibit *Pf*PanK1 also to inhibited *P. falciparum* erythrocytic stage parasites. However, while Gnetin C showed comparable IC_50_ values against *Pf*PanK and the erythrocytic stage parasites, diacetylkinamycin C, Simaomicin α, and Gnemonoside D showed >10 fold lower IC_50_ values against *P. falciparum* cells compared to those against the enzyme. Thus, the efficacy of diacetylkinamycin C, Simaomicin α, and Gnemonoside D toward malaria parasites can be due to off-target effects. Gnetin C showed relatively low toxicity toward human cell lines (Narayanan et al., 2015) and an acceptable selectivity index (SI) (6.6-6.8).

## Discussion

*Pf*PanK inhibitors were successfully identified using an assay system using recombinant *Pf*PanK in our study. In the previous studies (Saliba et al., 2005; Spry et al., 2005; Pett et al., 2015), the crude enzyme from *P. falciparum* lysates were used for enzymological studies and identification of inhibitors. The development of a high throughput screening system in our study has provided. It will allow *Pf*PanK inhibitors to be easily identified with no potential interference by parasite and host-derived factors.

*Plasmodium falciparum* has two putative PanK encoded by *PfPanK1* and *PfPanK2* genes. Both are transcribed in the blood stages, in which the steady state mRNA level of *PfPanK1* is 12-fold higher than that of *PfPanK2*. It was previously suggested that only *Pf*PanK1 is involved in the pantothenate-related metabolism in the blood stages of *P. falciparum* (Tjhin et al., 2018), because selection of resistant lines against a pantothenate analog led to mutations only in *PfPanK1*, but not *PfPanK2*, gene (Tjhin et al., 2018). Their study also suggests that *Pf*PanK1 is most likely essential for the intraerythrocytic proliferation of *P. falciparum.* Our observation that recombinant *Pf*PanK2 produced using the wheat germ cell-free expression system did not show PanK activity seems to agree to the previous reports (Hart et al., 2016; Tjhin et al., 2018). *Pf*PanK2 apparently lacks the ATP binding motif (Hart et al., 2016), which is needed in all kinases that contain the highly conserved P-loop or Walker A sequence motif (GXXXXGKT/S) (Obmolova et al., 2001). Thus, *Pf*PanK2 may serve as a scaffold for a protein complex containing *Pf*PanK1 or other components of the CoA biosynthesis, as suggested (Park et al., 2015; Hart et al., 2016). In any event, the lack of PanK activity of recombinant *Pf*PanK2 prompted us to conduct further studies on *Pf*PanK1.

*Pf*PanK1 can phosphorylate both pantothenate and pantetheine to generate 4-phosphopantothenate or 4-phosphopantethine using ATP or GTP as a phosphate donor. It was previously shown that in *P. falciparum* blood stage parasites, either pantothenate or pantetheine must be incorporated from the host cytoplasm because the biosynthetic pathways for pantothenate and pantetheine are lacking (Saliba et al., 1998). Incorporation of pantetheine allows a bypass of pantothenate to 4’-phosphopantetheine, by directly yielding 4’-phosphopantetheine from pantetheine by *Pf*PanK, as demonstrated by the growth rescue of both sexual and asexual stages by pantethine supplementation in the culture (Fletcher et al., 2016). 4’-Phosphopantetheine subsequently generates CoA by the last two enzymes, phosphopantetheine adenylyltransferase (*Pf*PPAT) and dephospho-CoA kinase (*Pf*DPCK). Using recombinant *Pf*PanK1 we demonstrated that *Pf*PanK1 phosphorylates both pantothenate and pantetheine, and thus can bypass a few initial steps of CoA biosynthesis in this parasite.

We have shown that *Pf*PanK1 is regulated by allosteric inhibition by CoA and acetyl CoA in a manner similar to other organisms (Calder et al., 1999; Brand and Strauss, 2005). Our previous report on *Entamoeba histolytica* PanK showed that CoA inhibits amebic PanK in a competitive manner with ATP and uncompetitive or non-competitive mode with pantothenate, while acetyl CoA seemed to inhibit it in a mixed fashion with both substrates (Nurkanto et al., 2018). It was shown that panthenol and a pantothenate analog hampered *in vitro* growth of *P. falciparum* erythrocytic stages by inhibition of pantothenate phosphorylation (Saliba et al., 2005; Spry et al., 2005; Fletcher and Avery, 2014). We have shown that panthenol unexpectedly increases *Pf*PanK1 activity *in vitro*. Therefore, 4’-phosphopanthenol, which is produced by phosphorylation of panthenol may inhibit the downstream enzymes including phosphopantothenoylcysteine synthetase (*Pf*PPCS) as previously proposed (Tjhin et al., 2018).

Many potential inhibitors that interfere with the CoA biosynthetic pathway in *Plasmodium* have been reported (Fletcher and Avery, 2014; Fletcher et al., 2016; Weidner et al., 2017). Although pantothenate analogs such as pantothenol (PanOH), CJ-15,801 (Saliba and Kirk, 2005; Saliba et al., 2005; Spry et al., 2005), N5-trz-C1-Pan, and *N*-PE-αMe-PanAm (Macuamule et al., 2015; Howieson et al., 2016) were expected to target PanK, they appeared to inhibit other enzymes than PanK. In the present study, we identified four best PanK inhibitors with the IC_50_ values being in a range of 20–50 µM from natural compounds by a robust enzyme-based assay using recombinant *Pf*PanK1. None of these inhibitors are pantothenate analogs and they represent new scaffolds as PanK inhibitors although the IC_50_ values are >20 µM. Therefore, the mode of inhibition from these four compounds is assumed to be either noncompetitive or uncompetitive, not competitive. However, the exact mechanisms of inhibition by these compounds need to be clarified in the future. It is equally plausible that these inhibitors directly block the phosphorylation of pantothenate and pantethine, which, in both cases, results in the depletion of 4’-phosphopantothenate for the downstream reaction mediated by *Pf*PPCS.

Gnetin C is a naturally-occurring stilbenoid, a dimer of resveratrol and its structurally related compounds, gnemonosides, which are originally isolated from the *Gnetum gnemon* seeds, an edible plant native to Southeast Asia (Aiso-Sanada et al., 2018). It was previously reported that gnetin-C was reported to possess anti-cancer properties (Espinoza et al., 2017), and it also increased the number of circulating natural killer (NK) cells in the immunomodulatory system (Nakagami et al., 2019). Diacetylkinamycin C belongs to the polyketide family of metabolites mainly produced by *Streptomyces*, and acts as an antitumor agent (Hasinoff et al., 2006; Ballard and Melander, 2008) and anti-gram-positive bacteria. Simaomicin α was originally produced by actinomycetes, *Actinomadura madura* subspecies *simaoensis*, and also reported to possess very potent antibiotic activity (Lee et al., 1989).

All selected *Pf*PanK1 inhibitors also inhibited blood stage *P. falciparum* cell *in vitro*. From four compounds, simaomicin α has the IC_50_ value against *P. falciparum* cell much lower than *Pf*PanK1 enzyme, more than 10,000-fold. It probably indicated that inhibition of *P. falciparum* cell is not or not only due to the inhibition of *Pf*PanK1. From previous report, this compound showed antimalarial activity *in vitro* and is known to be a cell-cycle effector in *P. falciparum* (Ishiyama et al., 2008), whereas the exact mechanism is unrevealed. in human cancer cell, target of simaomicin α has been described to suppress the retinoblastoma protein phosphorylation and promotes apoptosis (Koizumi et al., 2009). However, no molecular target has been identified from this compounds (Wang et al., 2013). Taken together, all four *Pf*PanK1 inhibitors identified in this study were confirmed have anti *P. falciparum* phenotypic and also have a broad range of activities.

## Conclusion

We established a high-throughput screening system against *P. falciparum* PanK, which catalyzes a rate-limiting step of the CoA biosynthesis, using the bacterial recombinant *Pf*PanK1. We also enzymologically characterized *Pf*PanK1. Finally, we identified four *Pf*PanK natural inhibitors with various scaffolds. The screening system is readily available for large chemically defined compound and microbial/plant extract libraries to discover new inhibitors. Further studies are needed to optimize the inhibitors to improve *Pf*PanK inhibitory activity.

## Data Availability Statement

The data presented in the study are deposited in the National Center for Biotechnology Information repository (https://www.ncbi.nlm.nih.gov/genbank/), accession number MW331581.

## Author Contributions

AN, the main contributor, designed and performed the experiments, analyzed the data, and wrote the manuscript. GJ designed the experiment and analyzed the data. HS performed the experiments and analyzed the data. YR, TS, and DI performed the experiments. MM and TN designed the experiments and analyzed the data. YN, KG, YS, and KS provided the chemical library and natural compounds. YT designed and performed the experiments and analyzed the data. TN conceived the project, acquired funding, wrote the manuscript, and supervised the study. All authors contributed to the article and approved the submitted version.

## Funding

This research is funded by Grants-in-Aid for Scientific Research (B) (KAKENHI JP18H02650 to TN) and JSPS-LIPI Joint Research Program (JPJSBP120208201 to TN) from the Japan Society for the Promotion of Science, Grant for research on emerging and re-emerging infectious diseases from Japan Agency for Medical Research and Development (AMED, JP19fk0108046 and JP20fk0108138 to TN), and Grant for Science and Technology Research Partnership for Sustainable Development (SATREPS) from AMED and Japan International Cooperation Agency (JICA) (JP19jm0110009 and JP20jm0110022 to TN).

## Conflict of Interest

The authors declare that the research was conducted in the absence of any commercial or financial relationships that could be construed as a potential conflict of interest.

## Supplementary Material

Supplementary Figure 1 Phylogenetic tree of PanK from *P. falciparum* and other species. Optimal ML tree was inferred by RAxML program with LG + Γ4 model is shown. 145 unambiguously aligned positions from 42 sequences were used for the analysis. Branch lengths are proportional to estimated numbers of substitutions. Bootstrap proportion (BP) values (shown in percentage) of higher than 50 are shown on the internal branches.

Supplementary Figure 2 Expression and solubility optimization of *Pf*PanK1 using *E. coli* expression system **(A)** Expression of *Pf*PanK1 in *E. coli* was induced with IPTG for 18 h. *Pf*PanK was equally well expressed in two independent bacterial clones (1 and 2). **(B)** *Pf*PanK1 solubilized in various concentrations of trehalose, examined on SDS-PAGE, followed by immunoblot analysis using anti-His-tag antibody. A red arrow head indicates expressed *Pf*PanK1 recombinant protein.

Supplementary Figure 3 Purification of recombinant *Pf*PanK1 in *E. coli* expression system. **(A)** Protein samples at each step of purification were subjected to 12% SDS-PAGE under reducing conditions, and the gel was stained with Coomassie Brilliant Blue. **(B)** Immunoblot analysis of recombinant *Pf*PanK1 at each purification step using anti-His-tag antibody.

Supplementary Figure 4 Specific activity of recombinant *Pf*PanK1 at various pHs. Assays were performed as described in Materials and methods in 15 mM HEPES, pH 8.3, 20 mM NaCl, 10 mM MgCl_2_, 1 mM EGTA, 0.02% Tween-20, 0.1 mg/mL β−globulins, and 50 ng of *Pf*PanK1 recombinant enzyme at 37°C. Data are shown in mean ± SEM of three replicates.

Supplementary Figure 5 Expression **(A)** and activity of recombinant *Pf*PanK2 **(B)**. **(A)** Expression of *Pf*PanK2 in wheat germ cell-free protein expression system. Red arrowheads indicate *Pf*PanK2. *Pf*PanK2-A and *Pf*PanK2-B represent lysates from two independent bacteria clones. GFP, INH2 and INH3 are positive controls (black and grey arrowheads). **(B)** PanK activities detected in lysates containing *Pf*PanK2. Assays were performed as described in Materials and methods, in a reaction of 100 µM ATP, 100 µM pantothenate in buffer contains 15 mM HEPES, pH 8.3, 20 mM NaCl, 10 mM MgCl_2_, 1 mM EGTA, 0.02% Tween-20, *Pf*PanK2 lysate, and 0.1 mg/mL β−globulins at 37°C. The data are shown in mean ± SEM of three replicates in arbitrary unit.

Supplementary Figure 6 Kinetic analysis of recombinant *Pf*PanK1. **(A)** The specific activity of *Pf*PanK1 at various pantothenate concentrations in the presence of 120 µM of ATP. **(B)** The specific activity of *Pf*PanK at various ATP concentrations in the presence of 100 µM of pantothenate. **(C)** The specific activity of *Pf*PanK at various pantetheine concentrations in the presence 120 µM ATP. **(D)** The specific activity of *Pf*PanK2 at various ATP concentrations in the presence of 100 µM pantetheine. Assays were performed with 15 mM HEPES, pH 8.4, 20 mM NaCl, 10 mM MgCl_2_, 1 mM EGTA, 0.02% Tween-20, 50 µg/mL *Pf*PanK, and 0.1 mg/mL β−globulins at 37°C. Data are shown in mean ± SEM of three replicates.

Supplementary Table 1 Purification of recombinant *Pf*PanK1. Enzyme activity was measured as described in Materials and methods.

Supplementary Table 2 Inhibitory activities (> 40%) of *Pf*PanK1 from natural products.
