# Supplementary material for: Characterization of Plasmodium falciparum Pantothenate Kinase and Identification of Its Inhibitors From Natural Products
Source: Front Cell Infect Microbiol. 2021 Mar 9;11:639065. doi: 10.3389/fcimb.2021.639065 (PMC7985445; doi:10.3389/fcimb.2021.639065)
Supplement: Supplementary Figure 1 — Phylogenetic tree of PanK from P. falciparum and other species. Optimal ML tree was inferred by RAxML program with LG + Γ4 model is shown. 145 unambiguously aligned positions from 42 sequences were used for the analysis. Branch lengths are proportional to estimated numbers of substitutions. Bootstrap proportion (BP) values (shown in percentage) of higher than 50 are shown on the internal branches. [file DataSheet_1.pdf]

Fig. S1

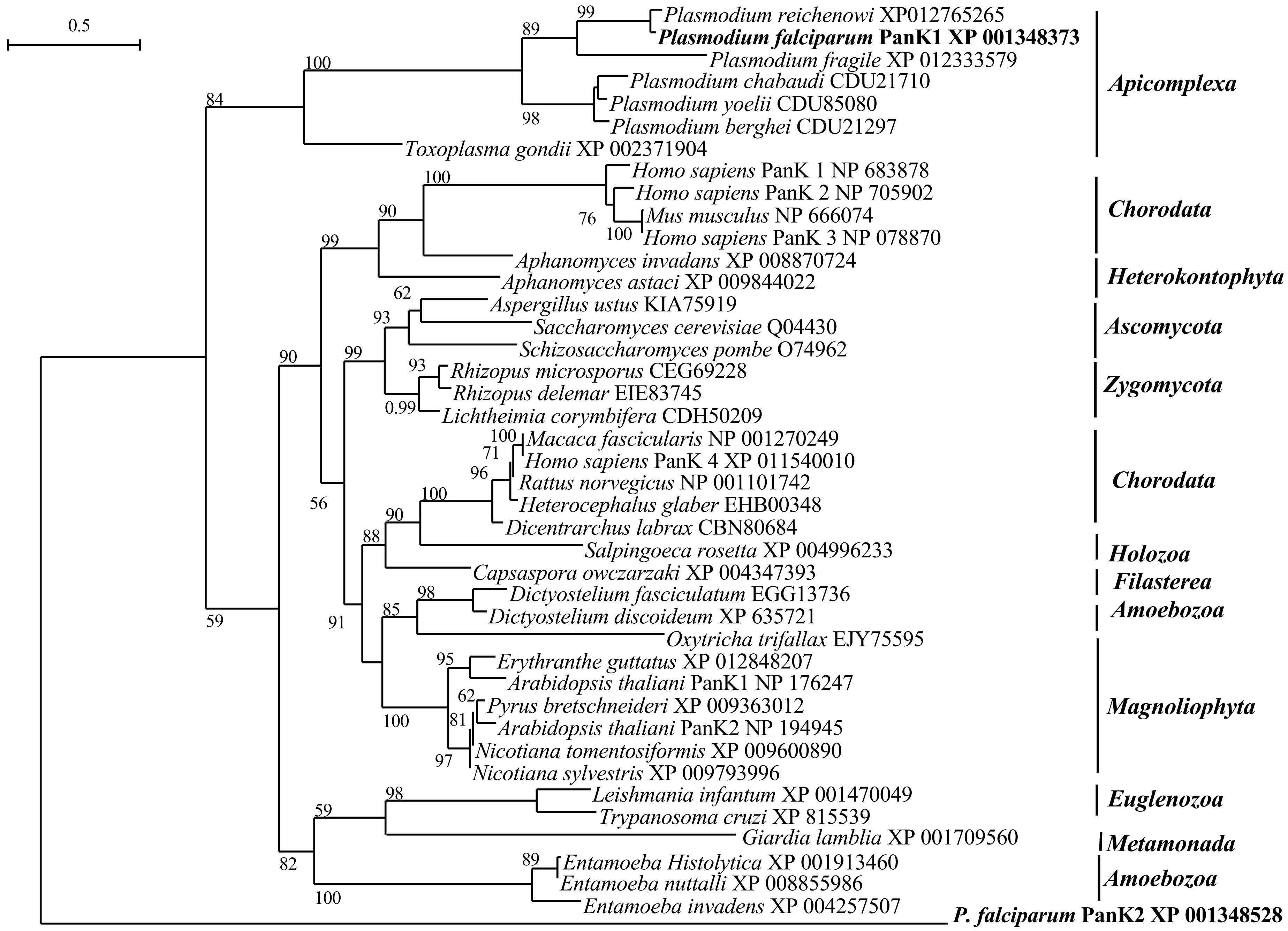
0.5
Plasmodium reichenowi XP012765265
Plasmodium falciparum PanK1 XP 001348373
Plasmodium fragile XP 012333579
Plasmodium chabaudi CDU21710
Plasmodium yoelii CDU85080
Plasmodium berghei CDU21297
Toxoplasma gondii XP 002371904
Homo sapiens PanK 1 NP 683878
Homo sapiens PanK 2 NP 705902
Mus musculus NP 666074
Homo sapiens PanK 3 NP 078870
Aphanomyces invadans XP 008870724
Aphanomyces astaci XP 009844022
Aspergillus ustus KIA75919
Saccharomyces cerevisiae Q04430
Schizosaccharomyces pombe O74962
Rhizopus microsporus CEG69228
Rhizopus delemar EIE83745
Lichtheimia corymbifera CDH50209
Macaca fascicularis NP 001270249
Homo sapiens PanK 4 XP 011540010
Rattus norvegicus NP 001101742
Heterocephalus glaber EHB00348
Dicentrarchus labrax CBN80684
Salpingoeca rosetta XP 004996233
Capsaspora owczarzaki XP 004347393
Dictyostelium fasciculatum EGG13736
Dictyostelium discoideum XP 635721
Oxytricha trifallax EJY75595
Erythranthe guttatus XP 012848207
Arabidopsis thaliani PanK1 NP 176247
Pyrus bretschneideri XP 009363012
Arabidopsis thaliani PanK2 NP 194945
Nicotiana tomentosiformis XP 009600890
Nicotiana sylvestris XP 009793996
Leishmania infantum XP 001470049
Trypanosoma cruzi XP 815539
Giardia lamblia XP 001709560
Entamoeba Histolytica XP 001913460
Entamoeba nuttalli XP 008855986
Entamoeba invadens XP 004257507
P. falciparum PanK2 XP 001348528
Apicomplexa
Chorodata
Heterokontophyta
Ascomycota
Zygomycota
Chorodata
Holozoa
Filasterea
Amoebozoa
Magnoliophyta
Euglenozoa
Metamonada
Amoebozoa
99
89
100
98
84
100
90
76
100
99
62
93
99
93
0.99
90
100
71
96
100
90
56
88
98
85
59
91
95
62
81
97
100
98
59
82
89
100

Fig. S2

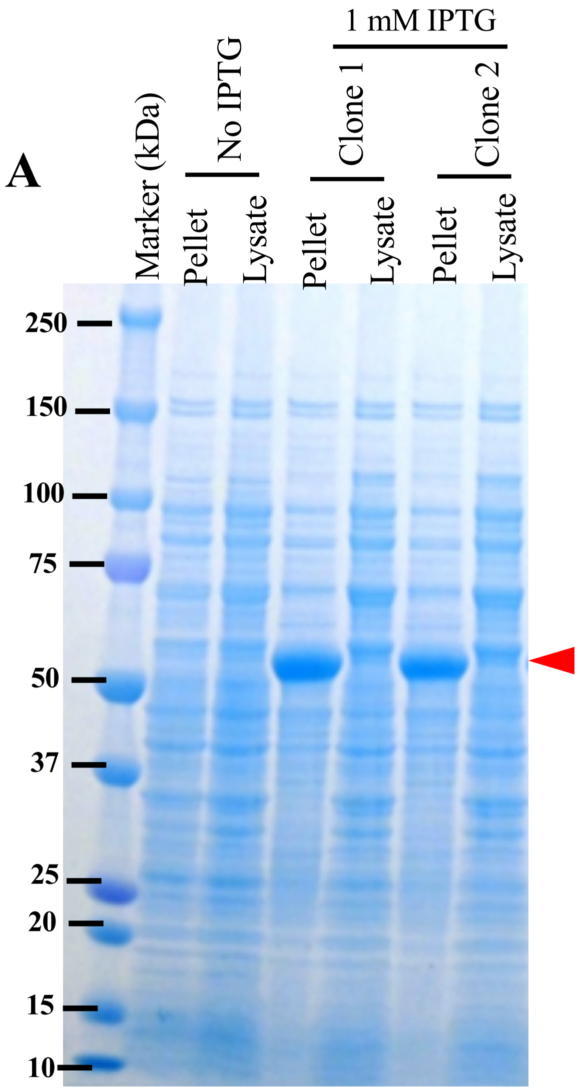
A
Marker (kDa)
No IPTG
1 mM IPTG
Clone 1
Clone 2
Pellet
Lysate
Pellet
Lysate
Pellet
Lysate
250
150
100
75
50
37
25
20
15
10

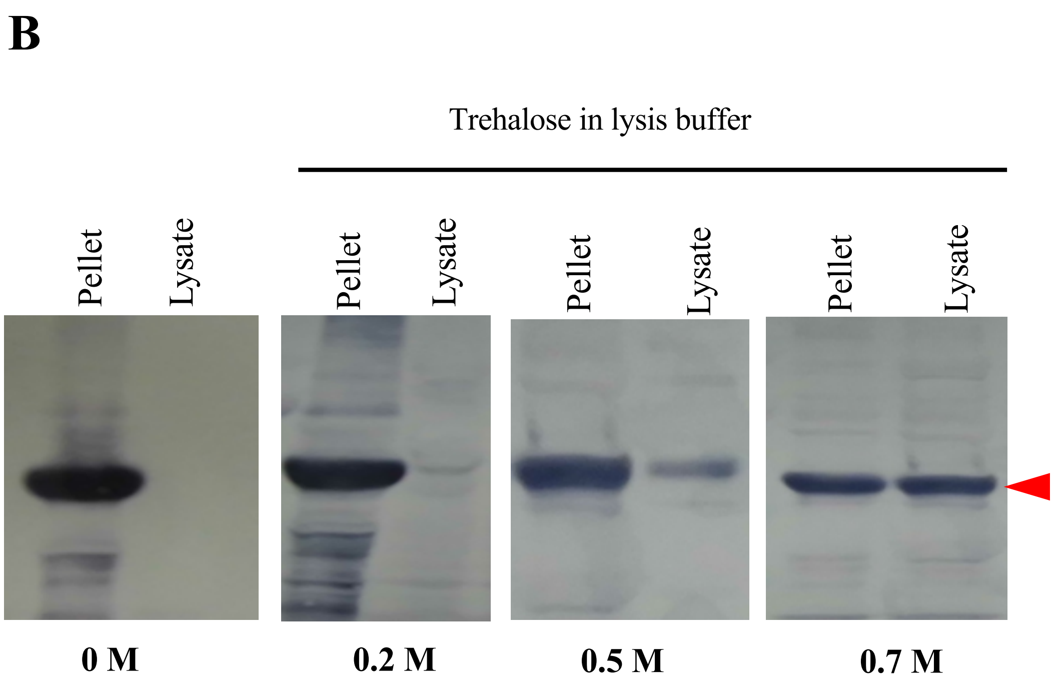
B
Trehalose in lysis buffer
Pellet
Lysate
Pellet
Lysate
Pellet
Lysate
Pellet
Lysate
0 M
0.2 M
0.5 M
0.7 M

Fig. S3

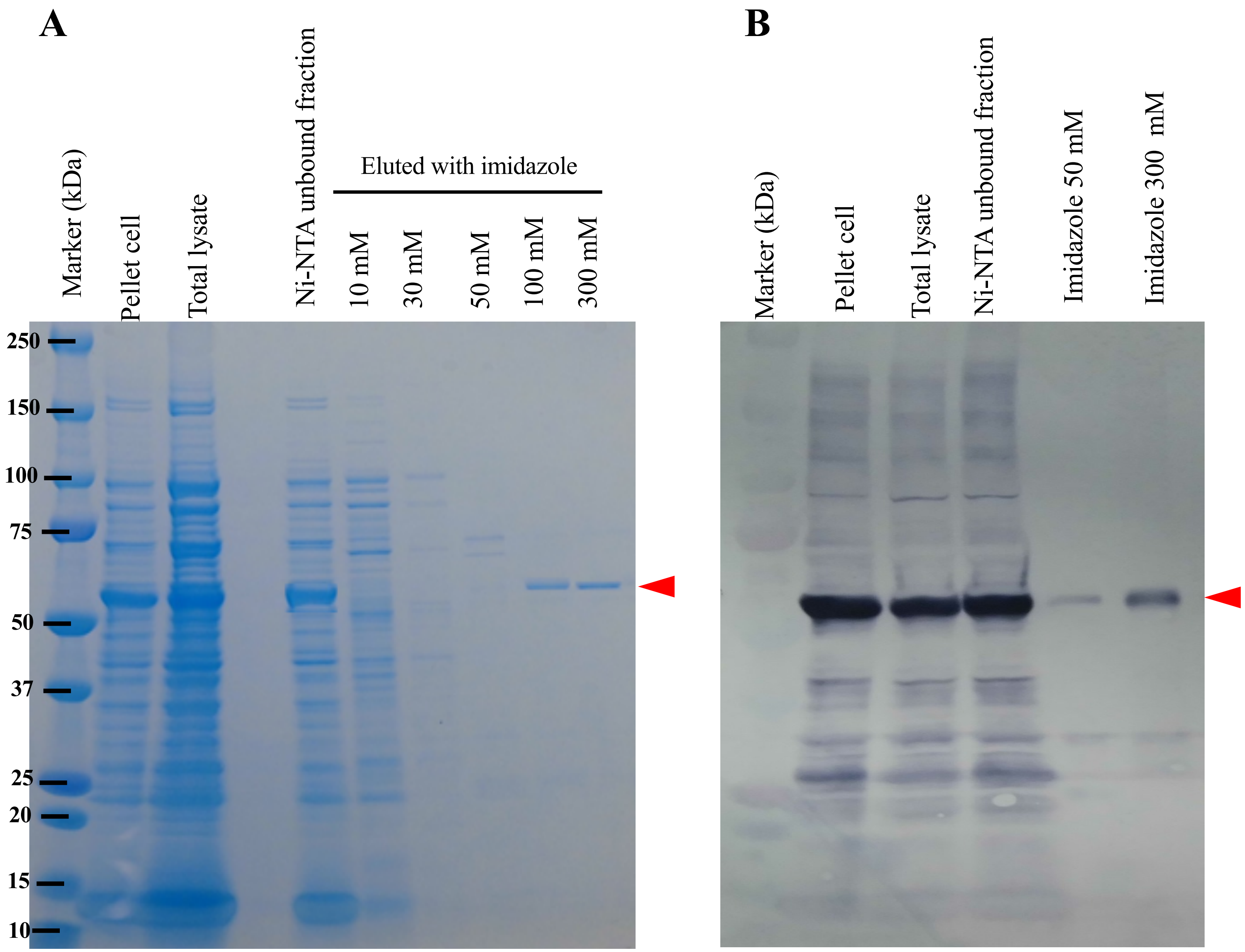
A
Marker (kDa)
Pellet cell
Total lysate
Ni-NTA unbound fraction
Eluted with imidazole
10 mM
30 mM
50 mM
100 mM
300 mM
250
150
100
75
50
37
25
20
15
10
B
Marker (kDa)
Pellet cell
Total lysate
Ni-NTA unbound fraction
Imidazole 50 mM
Imidazole 300 mM

Fig. S4

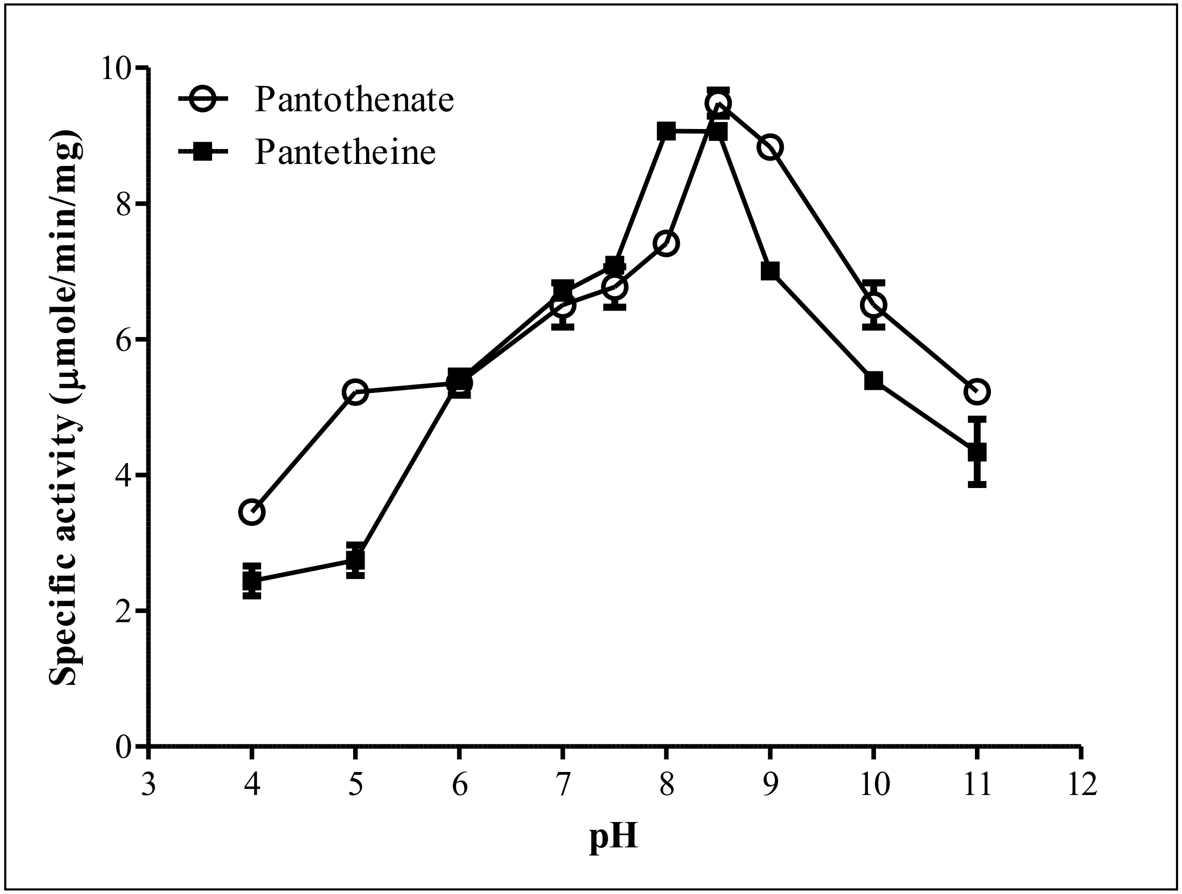

Pantothenate
Pantetheine
Specific activity (µmole/min/mg)
pH
0
2
4
6
8
10
3
4
5
6
7
8
9
10
11
12

Fig. S5

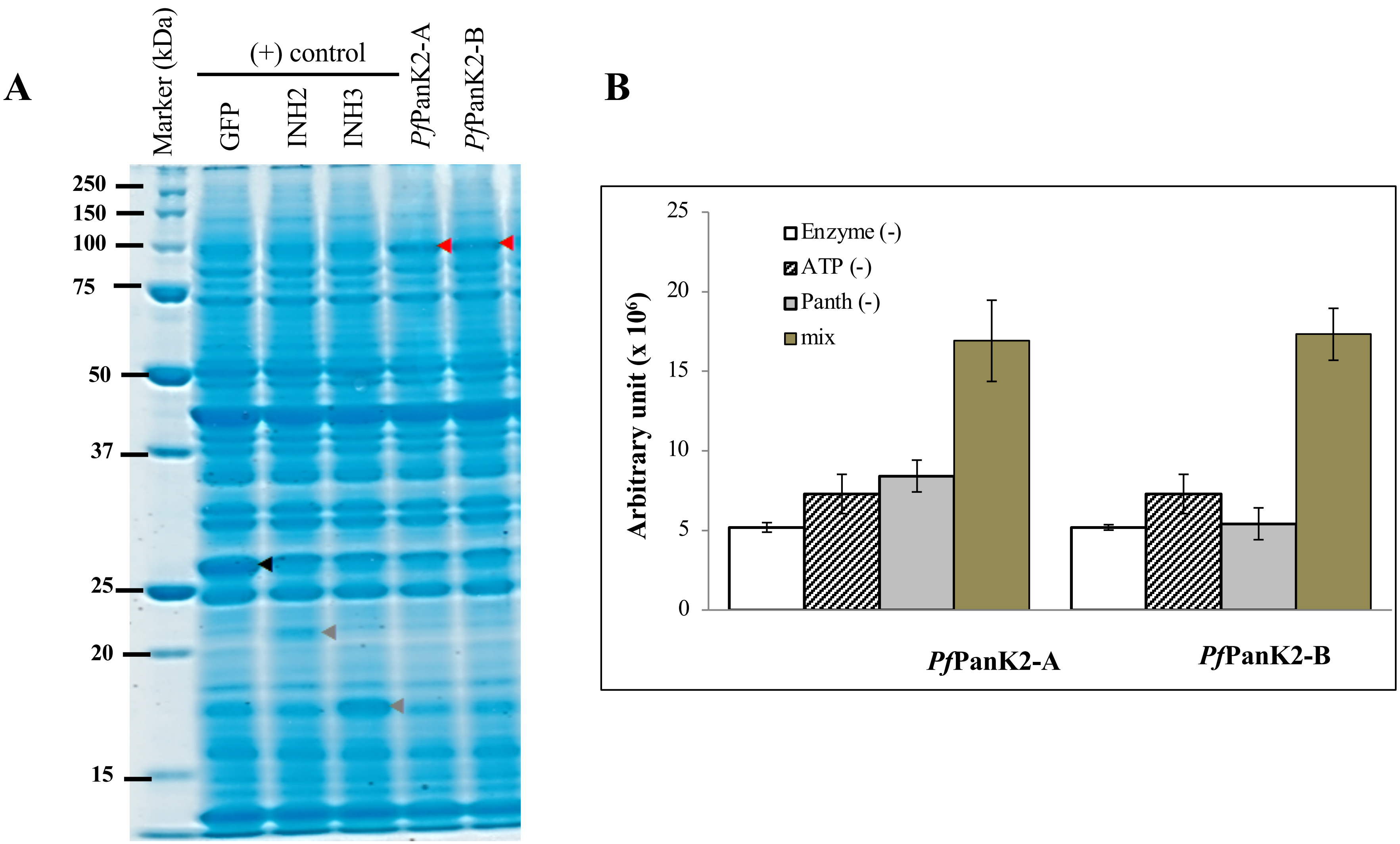
A
Marker (kDa)
(+) control
GFP
INH2
INH3
*Pf*PanK2-A
*Pf*PanK2-B
250
150
100
75
50
37
25
20
15
B
Arbitrary unit (x 10^6)
25
20
15
10
5
0
Enzyme (-)
ATP (-)
Panth (-)
mix
*Pf*PanK2-A
*Pf*PanK2-B

Fig. S6

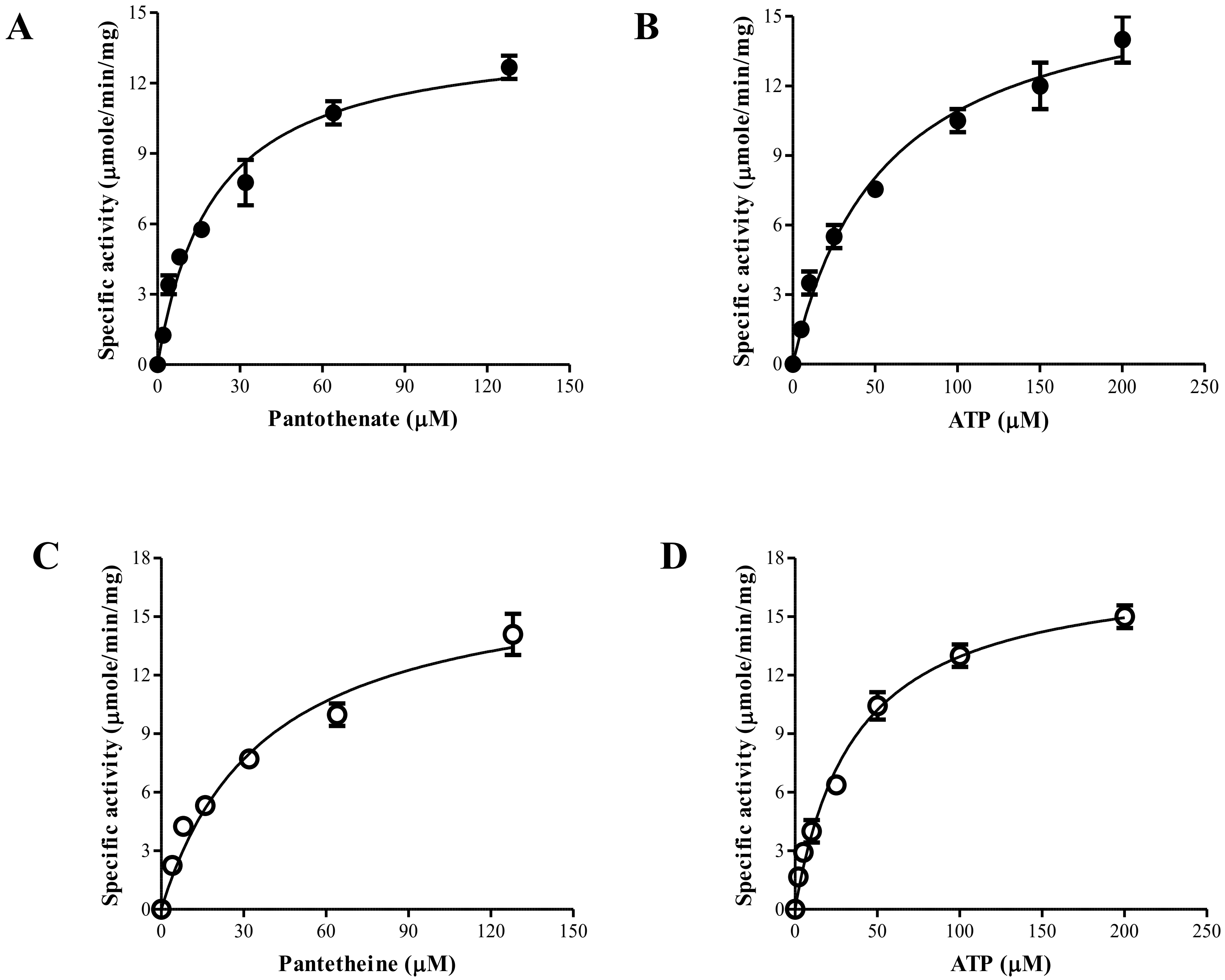
A
Specific activity (μmole/min/mg)
0
3
6
9
12
15
0
30
60
90
120
150
Pantothenate (μM)
B
Specific activity (μmole/min/mg)
0
3
6
9
12
15
0
50
100
150
200
250
ATP (μM)
C
Specific activity (μmole/min/mg)
0
3
6
9
12
15
18
0
30
60
90
120
150
Pantetheine (μM)
D
Specific activity (μmole/min/mg)
0
3
6
9
12
15
18
0
50
100
150
200
250
ATP (μM)

Table S1

Purification step	Protein content (mg)	Activity (µmole/min)	Specific activity (µmole/min/mg)	Yield (%)	Purification (fold)
Lysate	3.94 ± 0.18	3.51 ± 0.57	0.89 ± 0.14	100	1
Eluate	0.22 ± 0.02	2.13 + 0.01	9.66 ± 0.01	60.61+ 0.0	10.9 + 1.8

Table S2

Compound	*Pf*PanK1 Inhibition (%)
Diacetylkinamycin C	96.1
Gnetin C	89.8
Gnemonoside D	85.0
Simaomicin α	76.9
Spectinomycin (Actinospectacin)	55.0
Thaimycin	53.9
Aurodox	52.2
BA-17039-A (AM-3468)	51.3
Aspirochlorine	49.7
Tirandamycin A	49.7
Spectinomycin (Trobicin)	47.8
Trichostatin	47.2
Echinomycin	47.1
Ribostamycin	46.6
Amoxicillin	45.7
Gramicidin NF	45.5
Dihydrostreptomycin	44.7
3-AG	44.4
Cefazolin	44.2
Lividomycin A	44.0
Gardimycin	42.5
Thiolactomycin	42.4
Lincomycin	41.3
OM-173 αE	40.3
Leucomycin A3	40.1
